# Shape Memory Behavior of Carbon Black-reinforced Trans-1,4-polyisoprene and Low-density Polyethylene Composites

**DOI:** 10.3390/polym11050807

**Published:** 2019-05-06

**Authors:** Lin Xia, Han Gao, Weina Bi, Wenxin Fu, Guixue Qiu, Zhenxiang Xin

**Affiliations:** 1 Key Laboratory of Rubber-Plastics, Ministry of Education/Shandong Provincial Key Laboratory of Rubber-Plastics, School of Polymer Science and Engineering, Qingdao University of Science and Technology, Qingdao 266042, China; xialin@qust.edu.cn (L.X.); 13306383978@163.com (H.G.); biweina1981@163.com (W.B.); xinzhenxiang@126.com (Z.X.); 2 Materials Science and Engineering, School of Engineering, University of California at Merced, 5200 North Lake Road, Merced, CA 95343, USA; wfu7@ucmerced.edu

**Keywords:** trans-1,4-polyisoprene, low-density polyethylene, carbon black, shape memory, reinforce

## Abstract

Shape memory composites of trans-1,4-polyisoprene (TPI) and low-density polyethylene (LDPE) with easily achievable transition temperatures were prepared by a simple physical blending method. Carbon black (CB) was introduced to improve the mechanical properties of the TPI/LDPE composites. The mechanical, cure, thermal and shape memory properties of the TPI/LDPE/CB composites were investigated in this study. In these composites, the crosslinked network generated in both the TPI and LDPE portions acted as a fixed domain, while the crystalline regions of the TPI and LDPE portions acted as a reversible domain in shape memory behavior. We found the mechanical properties of composites were promoted significantly with an increase of CB content, accompanied with the deterioration of shape memory properties of composites. When CB dosage was 5 parts per hundred of rubber composites (phr), best shape memory property of composites was obtained with a shape fixity ratio of 95.1% and a shape recovery ratio of 95.0%.

## 1. Introduction

Shape memory polymers (SMPs) are smart stimuli responsive materials [1], which possess the capacity to fix temporary shapes and recover to their permanent shapes under external stimulus, such as moisture, heat, pH, light, electric and magnetic fields [2,3,4,5,6,7,8]. With merits of low manufacturing costs, tunable recovery temperature and possible biocompatibility, heat stimuli responsive SMPs have had extensive advances in both academic and industrial fields, which has granted this kind of smart material wide potential applications, such as in deployable aerospace structures, intelligent textiles, self-healing and biomedical devices [9,10,11,12,13,14].

Generally, SMPs are multiphase materials, which are comprised of fixed and reversible domains. Fixed domains are usually used to sustain permanent shapes by restricting plastic slippage of SMPs molecule chains, which could be hydrogen bonding, chain entanglement or chemical cross-linked points [15]. The crystalline regions or soft chain segment of polymers could work as reversible domains [16,17]. And reversible domains are utilized to obtain temporary shapes by freezing and unfreezing around transition temperatures (T_trans_) [18], which may be melting temperature (T_m_) for crystalline polymers or glass transition temperatures (T_g_) for amorphous polymers. Shape memory process of SMPs was described as follows [19]: First, the SMPs specimens were stretched at a temperature above T_trans_ with mechanical load applied. Second, stretched specimens were cooled at a certain rate without a mechanical load. The temporary shapes were generated and entropic elasticity was restored in the polymer matrix in this process. Finally, permanent shapes of SMPs were recovered when specimens were reheated above T_trans_, which was attributed to the entropic elasticity released from polymer matrix. The molecule chains with regular arrangement in specimens tended back to its random stages when specimens were reheated again.

Compared to other shape memory materials, such as shape memory ceramics (SMCs) and alloys (SMAs), SMPs exhibit excellent qualities as a high recoverable strain, controllable mechanical properties and easily shaped properties [20,21,22,23]. However, low recovery stress and poor mechanical properties of SMPs limit the applications of SMPs. Trans-1,4-polyisoprene (TPI) is a typical SMP when it is partly cross-linked, which is similar to cross-linked polyethylene (PE) [24,25]. The shape memory properties of TPI, PE and their blends have been investigated in recent years [26,27]. However, the disadvantage of most shape memory composites is their poor mechanical properties, and TPI and PE composites are no exception. Therefore, improving the mechanical properties of SMPs is a very important topic. Carbon black, as a common reinforcement filler, is widely utilized in the rubber industry with advantages of moderate pricing and extensive available sources [28,29,30,31,32]. Carbon black could also be used in conductive and functional sensing fields [33,34]. In this paper, carbon black (CB) reinforced shape memory composites of TPI and low-density PE (LDPE) were prepared by a mechanical blending method. The effect of CB on the mechanical, thermal and shape memory properties of composites were investigated in this manuscript. And a schematic diagram was proposed to illustrate the shape memory behavior of the TPI/LDPE composites. These kinds of composites are expected to be used as pipe connection materials, especially in heat shrinkable PE pipe connect parts.

## 2. Experimental

### 2.1. Materials

LDPE (type LD100AC) pellets with melt flow index (MFI) of 2.1 g (10 min)^−1^ (190 °C) were purchased from Yan-shan Petrochemical Co Ltd (Beijing, China). TPI powder (purity ≥ 99%) with a Mooney viscosity of 103.3 was purchased from Di Pai New Material Co Ltd (Qingdao, China). Carbon black (type N330) with an average particle diameter of 30 nm was purchased from Cabot Co Ltd (Shanghai, China). Rubber antioxidant 2-mercaptobenzimidazole (MB) was purchased from Xuzhou Lejin Chemical Technology Co Ltd (Xuzhou, China). Dicumyl peroxide (DCP) was purchased from Akzo Nobel polymer Chemicals (Ningbo, China). Other additives were obtained from commercial sources and were used without further purification.

### 2.2. Preparation of the TPI/LDPE/CB Composites

The LDPE, TPI and CB were dried in a vacuum oven at 50 °C for 6 h before use. The TPI/LDPE composites with different CB doses were prepared on a high-temperature open mill at 110 °C for 10 min with a standard mixing sequence. The composite formulations with different CB doses are shown in Table 1.

### 2.3. Cure Characteristics of the Composites

The cure characteristics of the composites were studied with a Monsanto oscillating disc rheometer (Monsanto Company, St. Louis, MO, USA) at 170 °C according to ASTM D-2084-07. The optimum cure time, scorch time and cure rate index were determined, and are presented in Table 2.

### 2.4. Mechanical Characterization

Vulcanized slabs were prepared by compression molding, and the dumbbell shaped specimens were punched out from a molded sheet by using an ASTM Die C (Wuxi KLT Precision Hydraulic Machinery Factory, Wuxi, China). The tests were conducted following ASTM D 412-06 and ASTM D 624-00 (2007). The modulus at 100% and 300%, elongation, tensile strength, tear strength and elongation at break were measured at room temperature. The initial length of the specimen was 25 mm, and the speed of the jaw separation was 500 mm min^−1^.

### 2.5. Differential Scanning Calorimetry (DSC)

The thermal properties of the blends were determined using a DSC-Q20 calorimeter (TA Instruments, New Castle, Delaware, USA) under a nitrogen atmosphere. The temperature and enthalpy were calibrated with an indium standard. Samples with a mass of 5–10 mg were maintained at 130 °C for 3 min to eliminate their thermal history before they were cooled to −50 °C at 10 °C min^−1^. The samples were subsequently heated to 130 °C at 10 °C min^−1^. Traces of the first cooling and second heating steps were recorded for analysis. The degree of crystallinity (X_c_) for each portion of sample was calculated by the following equation:

(1) $$Xc=\frac{\Delta Hm}{\Delta Hm^{*}}\times 100\%$$

where ΔH_m_ and ΔH_m_^*^ are the melting enthalpy of a certain polymer portion and its perfect melting enthalpy (ca. 186.8 J g^−1^ for TPI and ca. 277.1 J g^−1^ for LDPE), respectively.

### 2.6. Shape Memory Effect

The shape memory properties of the composites were analyzed by a DMA-Q800 instrument (TA Instruments, New Castle, Delaware, USA) in the Controlled Force’ mode. The preloading was 0.001 N, and the frequency was 1 Hz. The test samples with a thickness of 2.0 mm were cut into rectangular shapes with a width of 4.0 mm and length of 30.0 mm. The initial clamp gap was set to 6.0~8.0 mm. The heating and cooling rates were both 5 °C min^−1^. The procedures for determining the shape memory effect are described below.

First, the sample was maintained isothermally at 130 °C for 5 min to completely melt the crystalline regions of both TPI and LDPE (the initial strain was denoted ɛ_0_). Second, a mechanical load of 0.2 MPa was applied, and the sample was cooled to −20 °C at 5 °C min^−1^ to completely freeze the crystalline domain (ɛ_1,load_). After removal of the mechanical load, the sample was maintained at −20 °C isothermally for 5 min (ɛ_1_). Finally, the sample was reheated to 130 °C at 5 °C min^−1^ and maintained isothermally for 15 min (ɛ_0,rec_).

The shape fixity ratio (R_f_) and shape recovery ratio (R_r_) are crucial parameters for SME characterization. The R_f_ for the temporary shape 1 (R_f_ (0→1)) and the R_r_ for the recovery from temporary shape 1 to 0 (R_r_ (1→0)) are quantified as follows [35,36,37]:

(2) $$R_{f}(0\to 1)=\frac{\varepsilon_{1}-\varepsilon_{0}}{\varepsilon_{1}{}_{,load}-\varepsilon_{0}}\times 100\%$$

(3) $$R_{r}(1\to 0)=\frac{\varepsilon_{1}-\varepsilon_{0}{}_{,rec}}{\varepsilon_{1}{}_{}-\varepsilon_{0}}\times 100\%$$

where ɛ_load_ represents the maximum strain under the load, ɛ is the strain after cooling and load removal, and ɛ_rec_ is the recovered strain.

## 3. Result and Discussion

### 3.1. Curing, Mechanical Properties

First, we studied the effect of the CB dose on cure characteristics (Table 2). Dicumyl peroxide (DCP) is a peroxide vulcanizing agent, which not only vulcanized TPI containing double bonds, but also crosslinked LDPE without double bonds. Therefore, under the action of DCP, the composite would form a TPI cross-linking phase, a LDPE cross-linking phase, a TPI and a LDPE co-cross-linking phase. The scorch time (T_10_) and optimum cure time (T_90_) were gradually shortened as the CB dose was increased. The values of M_H_-M_L_, the reflection of crosslinking extent of composites, increased with an increasing CB content. Besides the generation of cross-link network under the fixed dosage of DCP, the aggregation of CB particles was also responsible for melt viscosity improvement of composites. The baffle effect on melt viscosity of TPI/LDPE composites was amplified by the massive CB dosage. Meanwhile, oxygen-containing groups (such as hydroxyl and carboxyl) on the surface of CB particles might act as an accelerator agent in the cross-linking reaction. Therefore, the scorch time T_10_ and optimum cure time T_90_ of composites were shortened as CB dosage increased.

Next, we tested the mechanical properties of the composites (Table 3). We found that all mechanical performance indexes were improved notably by CB incorporation. Especially, when the carbon black content was 15 phr, the tensile strength and tearing strength of composites were increased about 5 MPa and 10 kN/m, respectively. We speculated that chemical and physical cross-linkages were both contributed to this phenomenon. Chemical cross-linkage, which generated by DCP, provided chemical bonding for molecule chains of the polymer matrix. But this enhancement effect remained constant due to the fixed DCP content. The physical cross-linkage generated by interactions between CB particles and molecule chains of polymer matrix promoted the mechanical properties of composites, which became excellent with the increase of CB content.

### 3.2. DSC Analysis

**Figure 1:**
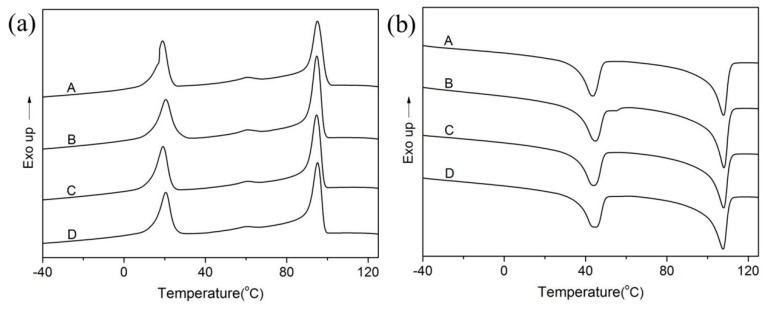
Effect of CB dosage on DSC curves of TPI/LDPE composites, (**a**) cooling curves and (**b**) heating curves.

Both TPI and LDPE easily crystallize due to their regular macromolecular chain structures. The addition of CB would affect not only the mechanical properties but also the thermal properties of the materials. Therefore, it is very important to investigate the effect of CB on the crystallization of composites. Both the thermal properties and transition temperatures of the shape memory composites can be measured by DSC. The effect of CB dosage on melting temperature (T_m_), peak position and crystallinity of composites were investigated and shown in Figure 1 and Table 4. We found the peak positions for melting and crystallizing temperature remained almost constant, which might be attributed to the fixed dosage of cross-link agent (DCP). However, melting enthalpies for each portion of composites were elevated slightly then decreased as CB dosage increased. The increase of melting enthalpies might be due to the crystallization nucleation effect of CB in composites. However, the flexibility of polymer molecular chains would be impaired leading to the reduction of crystallinity degree of TPI and LDPE portions when CB dosage reached to high value.

### 3.3. Shape Memory Effect Analysis

**Figure 2:**
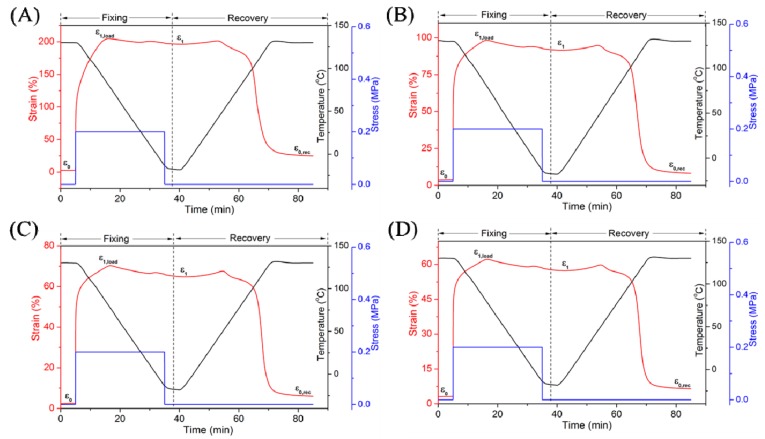
Effect of CB dosage on shape memory properties of TPI/LDPE blends.

Finally, shape memory properties of TPI/LDPE/CB composites were measured by a dynamic mechanical analyzer (Figure 2 and Table 5). Specimens of SMPs was kept isothermally above T_trans_ (130 °C). The crystalline regions of TPI and LDPE were both melted, which acted as reversible domains in composites. Meanwhile, the cross-linking network acted as fixed domain to restrict plastic slippage of molecular chains. We found the addition of CB could improve the shape memory recovery ratio partly. The shape recovery ratio (R_r_) of composites was improved from 88.6% to 95.0% with the addition of CB filler (5 phr). The shape fixity ratio (R_f_) of composites changed little and kept above 91%. The improvement of R_r_ was due to the physical network generated by introducing CB, which provided additional entropic elasticity restored in specimens during the shape memory test. Meanwhile, crystalline property of TPI/LDPE composites were improved by adding a small amount of CB (5 phr), which made proper proportion between fixed and reversible domains. The formation of CB physical network and increase of crystallinity of polymer phase resulted in the improvement of shape memory property of composites. However, R_f_ and R_r_ showed decreasing tendencies, when CB dosage was elevated again. Obviously, relatively high dosages of CB (10 phr and 15 phr), accompanied with enhancement of physical cross-linkage, was harmful for reservation of the shape memory effect (SME) by producing a low crystallinity of polymer matrix and bad chain flexibility. However, the addition of CB improved the total crystallinity of TPI and LDPE components in composites (Table 4). Therefore, the addition of a large dose of CB still improved the shape memory properties of composites compared with those without CB.

**Figure 3:**
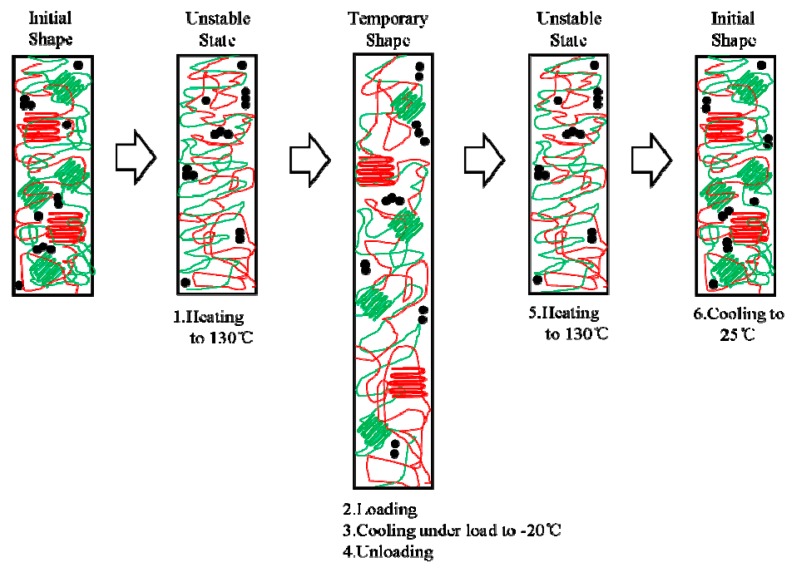
Schematic diagram of shape memory effect for TPI/LDPE/CB composites.

To make distinct comprehensions of the shape memory process of TPI/LDPE/CB composites, a schematic diagram was proposed in Figure 3. Green and red colors represent TPI and LDPE portions, respectively. Meanwhile, lines with disorder and regular distribution stand for amorphous and crystalline regions of TPI/LDPE/CB composites, respectively. Black circles represent aggregation of CB particles. Crystalline regions of TPI/LDPE/CB composites were melted completely when samples were kept isothermally at 130 °C for 5 min. Mechanical load was applied and temperatures were cooled down to −20 °C, the temporary shape was formed and fixed. After the load removal and reheating process, specimens were returned to their permanent shapes again.

## 4. Conclusions

Shape memory composites of trans-1,4-polyisoprene (TPI) and low-density polyethylene (LDPE) with easily achievable transition temperatures were prepared by a simple physical blending method. Carbon black (CB) was introduced to improve the mechanical properties of the TPI/LDPE composites. The mechanical, cure, thermal and shape memory properties of the TPI/LDPE/CB composites were investigated in this study. In these composites, the crosslinked network generated in both the TPI and LDPE portions acted as a fixed domain, while the crystalline regions of the TPI and LDPE portions acted as a reversible domain in shape memory behavior. We found the mechanical properties of composites were promoted significantly with an increase of CB content, accompanied with the deterioration of shape memory properties of composites. When CB dosage was 5 phr, the best shape memory property of composites was obtained with a shape fixity ratio of 95.1% and a shape recovery ratio of 95.0%. However, the mechanical properties of TPI/LDPE composites reached their peak values when the CB dosage was 15 phr, of which the shape fixity ratio was 91.8% and the shape recovery ratio was 93.9%, respectively.

## Figures and Tables

**Table 1:** Formulations of TPI/LDPE/CB composites (parts per hundred of rubber).

Chemicals	A	B	C	D
TPI	60	60	60	60
LDPE	40	40	40	40
DCP	0.4	0.4	0.4	0.4
Antioxidant MB	1.5	1.5	1.5	1.5
CB (N330)	0	5	10	15

**Table 2:** Effect of carbon black (CB) dosage on cure characteristics of TPI/LDPE composites.

Properties	Dosage of CB (N330)			
	A	B	C	D
M_H_ (dN·m)	4.95	5.79	6.66	7.61
M_L_ (dN·m)	2.06	2.42	2.68	2.99
M_H_-M_L_ (dN·m)	2.89	3.37	3.98	4.62
T_10_ (min)	1.30	1.15	1.07	1.03
T_90_ (min)	16.00	13.67	13.02	11.83
Cure rate index (min^−1^)	6.80	7.99	8.37	9.26

**Table 3:** Effect of CB dosage on mechanical properties of TPI/LDPE composites.

Properties	Dosage of CB (N330)			
	A	B	C	D
Tensile strength (MPa)	14.9	17.3	18.5	19.8
100% modulus (MPa)	7.4	8.3	8.4	8.6
300% modulus (MPa)	9.7	11.4	11.7	12.5
Elongation at break (%)	575	567	602	612
Tear strength (kN/m)	69.2	76.2	78.2	79.4

**Table 4:** Effect of CB dosage on crystallinity of TPI/LDPE composites.

Properties	Dosage of CB (N330)			
	A	B	C	D
X_c (TPI)_ (%)	12.3	14.4	13.7	12.7
T_m (TPI)_ (°C)	35.19	35.45	34.95	35.61
ΔH_m(TPI)_ (J/g)	22.91	26.91	25.62	23.78
X_c (LDPE)_ (%)	13.4	14.7	14.4	13.1
T_m (LDPE)_ (°C)	99.87	100.40	99.46	99.02
ΔH_m(LDPE)_ (J/g)	37.02	40.79	39.89	36.37
X_c (TPI__+LDPE)_ (%)	25.7	29.1	28.1	25.8

**Table 5:** Effect of CB dosage on shape memory properties of TPI/LDPE composites.

Properties	Dosage of CB (N330)			
	A	B	C	D
R_f_ (%)	95.7	95.1	92.0	91.8
R_r_ (%)	88.6	95.0	94.1	93.9
